# Reducing the harm associated in treating hyperkalaemia with insulin and dextrose

**DOI:** 10.1016/j.clinme.2024.100222

**Published:** 2024-06-12

**Authors:** Sara Abou Sherif, Irene Katsaiti, Hannah Jebb, Serena Banh, Rachna Bedi, Jeremy Levy, David Thomas, Damien Ashby, Richard Corbett

**Affiliations:** a Renal and Transplant Centre, Hammersmith Hospital, Imperial College Healthcare NHS Trust, London, United Kingdom; b Chelsea and Westminster Hospital, Chelsea and Westminster Hospital NHS Foundation Trust, London, United Kingdom; c Jeffrey Cheah Biomedical Centre, University of Cambridge, Cambridge, United Kingdom

**Keywords:** Hyperkalaemia, Hypoglycaemia, Insulin, Sodium zirconium cyclosilicate, Guideline

## Abstract

Inpatient treatment of hyperkalaemia with insulin and dextrose can be complicated by iatrogenic hypoglycaemia. We sought to assess the incidence of hypoglycaemia in hospitalised patients with renal disease and assess the impact of the introduction of a local guideline incorporating the use of sodium zirconium cyclosilicate (SZC) for patients with moderate hyperkalaemia. After establishing a significant burden of hypoglycaemia in the initial observation period, a requirement for hourly capillary blood glucose monitoring (for up to 6 h) following the administration of insulin for hyperkalaemia was incorporated into the guidelines. The two-fold introduction of SZC alongside changes in patient care after the administration of insulin/dextrose resulted in more appropriate use of insulin/dextrose, as well as a significant (73%) reduction in the iatrogenic burden of hypoglycaemia (*P* = 0.04).

## Introduction

Hyperkalaemia (serum potassium >5.5 mmol/L) is a common electrolyte disorder that is associated with the risk of cardiac arrhythmia and cardiac arrest.^1^^,^^2^ Insulin-dextrose (insulin/dextrose) is a common first-line treatment for both moderate hyperkalaemia (6.0–6.4 mmol/L) with evidence of potential cardiac instability, and severe hyperkalaemia (potassium > 6.5 mmol/L).^2^ Insulin stimulates activation of the sodium–potassium ATPase pump, leading to potassium influx into cells.^3^ Patients with renal dysfunction are at a significantly increased risk of hyperkalaemia.^4^

Insulin/dextrose treatment can be complicated by hypoglycaemia (serum glucose <4.0 mmol/L) in 6–21% of people following treatment.^1^, ^2^, ^3^, ^4^, ^5^, ^6^, ^7^ Hypoglycaemia poses significant risk to patients, including increased risk of seizures, neurological symptoms and cardiac instability, with associated morbidity and ultimately mortality.^5^ The risk of hypoglycaemia is greatest in those with low body mass and impaired renal function.^3^^,^^4^^,^^8^ The precise window following insulin/dextrose in which hypoglycaemia occurs remains debated; as a result, blood glucose monitoring varies across institutions.^3^

In attempts to avoid the morbidity associated with insulin/dextrose-related hypoglycaemia, a range of approaches have been attempted with varying efficacy.^1^ For decades, potassium binders have been utilised as adjuncts for treating acute hyperkalaemia. More recently, sodium zirconium cyclosilicate (SZC), a highly selective cation exchanger in the gastrointestinal tract, has been licensed for use in acute hyperkalaemia.^9^

The aim of this project was two-fold: firstly, to assess the incidence of hypoglycaemia in hospitalised patients with renal disease following acute treatment of hyperkalaemia with insulin and dextrose. Secondly, to assess the impact of the subsequent introduction of a local guideline incorporating the use of sodium zirconium cyclosilicate (SZC) for patients with moderate hyperkalaemia, on iatrogenic hypoglycaemia.

## Methods

### Study population

This was a single-centre quality improvement project (QIP), performed in a large urban renal centre using retrospectively collected data obtained from the electronic patient record. To assess the incidence of hypoglycaemia, all patients who received insulin/dextrose under the care of a renal physician were identified across 6 months from January to June 2019. The index local guideline required IV administration of 10 international units of a fast-acting insulin delivered parenterally as an infusion with 100 mL 20% glucose. Patients who received insulin/dextrose for reasons other than hyperkalaemia were excluded. All patients with renal disease, regardless of modality of renal replacement therapy, were included.

### Data collection

Electronic medical records were used to determine patient demographics (age and sex) and specific comorbidities relevant to hypoglycaemia risk (diabetes and renal replacement therapy modality). The medical records were examined to confirm the pre-treatment serum potassium level and any relevant peri-treatment electrocardiogram (ECG) monitoring, as well as any changes that might be attributable to hyperkalaemia (peaked T waves, P wave flattening/disappearance, PR interval elongation, QRS prolongation, conduction abnormalities or arrythmias). Hyperkalaemia was defined as mild (5.5–5.9 mmol/L), moderate (6.0–6.4 mmol/L) and severe (≥6.5 mmol/L). Patient records were scrutinised for pre-treatment glucose level and subsequent blood glucose levels in the 24 h following insulin/dextrose administration. The primary outcome, hypoglycaemia, was defined as mild (glucose ≤4.0 mmol/L) or severe (≤2.8 mmol/L), regardless of symptoms and requirement for intravenous, intramuscular or oral glucose replacement. This definition included blood glucose levels tested by either plasma or capillary blood glucose sampling; the timing of the nadir of blood glucose levels in the 24 h following insulin/dextrose treatment was also identified. All capillary blood glucose monitors automatically report directly into the electronic patient record.

### Interventions

Following a review of the initial cycle with multidisciplinary involvement, local guidelines were rewritten to encompass two key changes. Firstly, given the recent licensing of SZC, this was incorporated for use in the treatment of moderate hyperkalaemia in the absence of ECG changes. Guidance included the option for physicians to start SZC 10 g three times a day for up to 72 h. This could then be stopped once serum potassium fell into the normal range, typically within 24–48 h. Cautions for use of SZC included advice to administer it 2 h before or after azole antifungals, anti-retrovirals and tyrosine kinase inhibitors. Secondly, based on the median time to hypoglycaemia, the guidelines identified the need for capillary blood glucose monitoring up to 6 h post-insulin/dextrose treatment in all patients. Healthcare professionals were also advised to consider an additional glucose infusion of 250 mL 10% glucose over 6 h, in those who were lean, frail, older, women or had impaired kidney function. The updated guideline was disseminated widely through both educational sessions and audit meetings to the multidisciplinary team.

A second cycle of data collection was undertaken over 6 months between January and June 2021 (6 months after the introduction of the new guidelines) using identical criteria, but also included all patients in whom SZC was initiated for hyperkalaemia. Patients who were already on a maintenance SZC at the time of hyperkalaemia were excluded. We also excluded patients where SZC was used in conjunction with other potassium-lowering interventions such as insulin/dextrose. We recorded the length of treatment (hours) and time to normalisation of serum potassium (<5.5 mmol/L).

### Statistical analysis

Normally distributed data are reported as mean ± standard deviation while non-normally distributed data reported as median (interquartile range). Independent-sample *T* test and Fisher's exact *T* test were used to determine differences between the numerical and categorical variables, between groups, respectively; *P*-values <0.05 were considered statistically significant. Associations with hypoglycaemia, established in previous studies; age, renal dysfunction and diabetes^3^^,^^4^ were explored by logistic regression and odds ratios (ORs) with associated 95% confidence intervals (CIs).

## Results

### Frequency of insulin dextrose use for hyperkalaemia

During the first observation period (first 6 months of 2019), insulin/dextrose was administered for hyperkalaemia on 126 occasions (75 patients, median age 58 (46–67) years), during which time the renal unit experienced a total of 843 admissions. In comparison, during the second period (first 6 months of 2021) 6 months after guideline introduction (‘the intervention’), insulin/dextrose was used on 21 occasions (21 patients, median age 58 (42–69) years) despite a comparable denominator of 780 total admissions]. The demographics of the population treated with insulin/dextrose for hyperkalaemia were similar in both time periods (Table 1).

**Table 1:** Patient characteristics two treatment periods 2019 (pre-intervention) and 2021 (post-intervention).

	2019 pre-intervention *N = 75 patients	2021 post-intervention ⁎⁎*N* = 79 patients	*P* value
**Age, median (interquartile range), years**	58 (46–67)	60 (45–69)	0.4
**Male, *no. (%)***	48 (64)	39 (49)	0.08
**Diabetes mellitus, *no. (%)***	39 (52)	39 (49)	0.8
**Renal replacement *no. (%)***			
Haemodialysis	32 (43)	36 (46)	0.3
Peritoneal dialysis	6 (8)	8 (10)	
Kidney transplant	25 (33)	16 (20)	
CKD not on renal replacement therapy	12 (16)	19 (24)	

*Abbrevations:* CKD; chronic kidney disease.

⁎ *N* = total number of patients in the 2019 receiving acute treatment for hyperkalaemia (insulin/dextrose).

⁎⁎ *N* = total number of patients in 2021 receiving acute treatment for hyperkalaemia (insulin/dextrose (21 patients) and SZC (58 patients)).

### Incidence of hypoglycaemia

During the first observation period, 34% (42/124) of insulin/dextrose treatments resulted in hypoglycaemia, of whom 17/42 developed severe hypoglycaemia (Table 2). Two patients were excluded from the glucose analysis due to no blood glucose monitoring. All patients in 2021 had capillary blood glucose testing on at least one occasion after insulin/dextrose administration. Across all patients who experienced hypoglycaemia, there was no significant difference in the distribution of diabetes; however, a greater proportion of female patients experienced hypoglycaemia. Time to lowest glucose level among those experiencing hypoglycaemia was 3 (2–7) h.

**Table 2:** Breakdown of patient characteristics, insulin/dextrose treatment and glucose levels in the two treatment periods 2019 (pre-intervention) and 2021 (post-intervention).

	2019 pre-intervention *N* = 75 patients	2021 post-intervention *N* = 79 patients
**Total no of admissions**	843	780
Treatment no. (%) administrations*		
Insulin/dextrose Sodium zirconium cyclosilicate	126 (100) 0 (0)	21 (27) 66 (73)
**Insulin Dose**⁎⁎		
*Short-acting insulin dose n (%)* 5 Units 10 Units	7 (5) 119 (95)	0 (0) 21 (100)
**Insulin/dextrose dose in last 24 h** **n (%)**	34 (27)	0 (0)
**Hypoglycaemia**⁎⁎⁎		
None Mild Severe <2.8	82 (65) 25 (20) 17 (13)	71 (96) 3 (4) 0 (0)
**Glucose monitoring**		
Glucose checked 1° prior to treatment with insulin/dextrose. *no. (%)*	80/126 (63)	44/87 (51)
Trough blood glucose in 24 h, mmol/L, median (interquartile range)	5.3 (3.7–6.9)	6.1 (4.0–8.9)
Time to trough glucose, hours, median (interquartile range)	4.0 (2.0–15.0)	9 (3.8–19.0)

⁎ Expressed as percentage of total number of patients.

⁎⁎ Expressed as a percentage of total number of administrations of insulin/dextrose or sodium zirconium cyclosilicate.

⁎⁎⁎ Expressed as percentage of total number of administrations with subsequent blood sugar monitoring. In 2019, of the 126 insulin/dextrose administrations 2 did not have blood sugar monitored, therefore hypoglycaemia calculated as percentage of 124. In 2021, hyperkalaemia was treated with insulin/dextrose on *n* = 21 occasions and sodium zirconium on *n* = 66 occasions. In 53 of 66 SZC administrations, at least one blood sugar was recorded within 24 h of starting treatment, therefore hypoglycaemia calculated as percentage of total of 74.

**Fig. 1:**
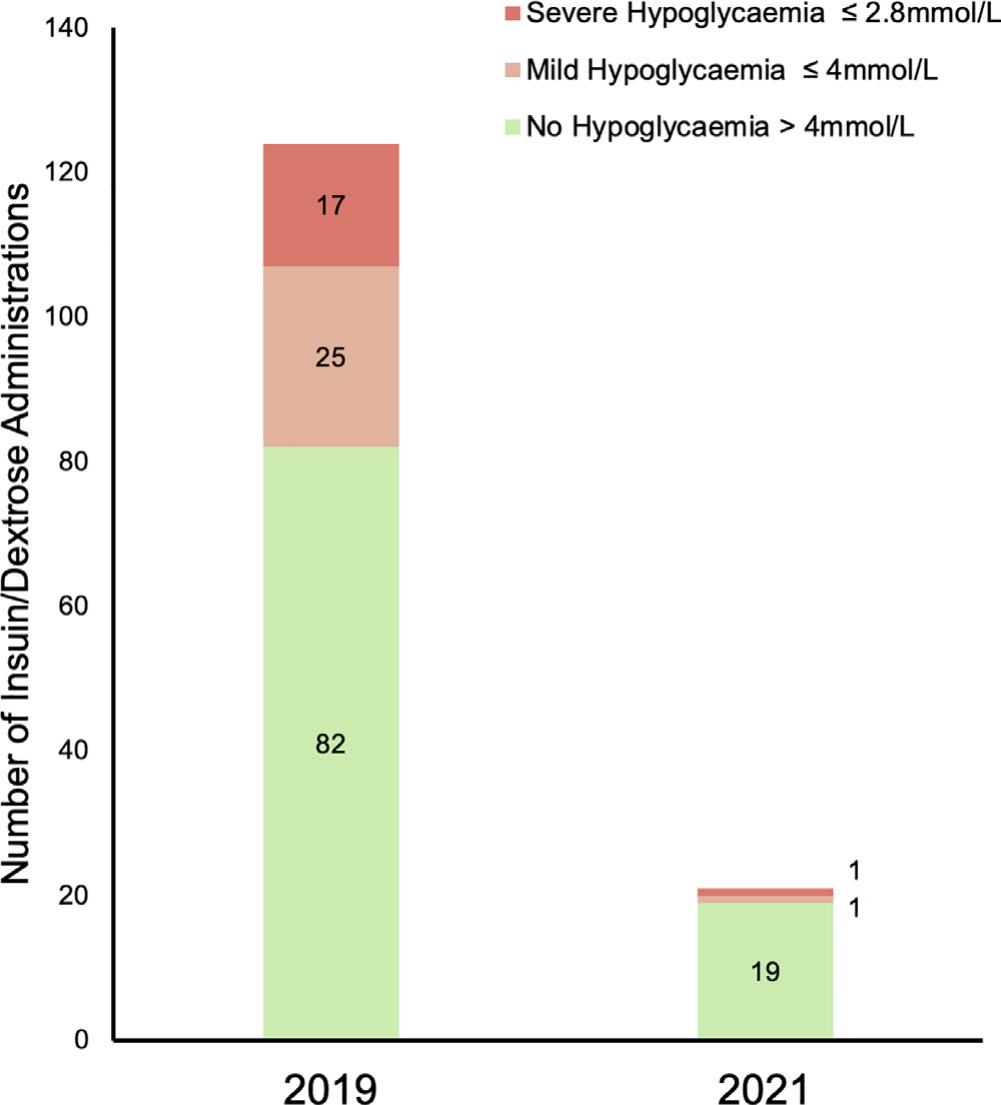
Bar chart illustrating the incidence of hypoglycaemia 24 h following administration of insulin/dextrose in 2019 and 2021.

Following the introduction of the new guidelines, not only was there a reduced usage of insulin/dextrose but in those who did receive insulin/dextrose, there was a 73% reduction in the incidence of hypoglycaemia (*P* = 0.04) (Fig. 1). Hypoglycaemia occurred in 9.5% (2/21) episodes, of which one was severe.

### Improved use of insulin dextrose treatment

**Fig. 2:**
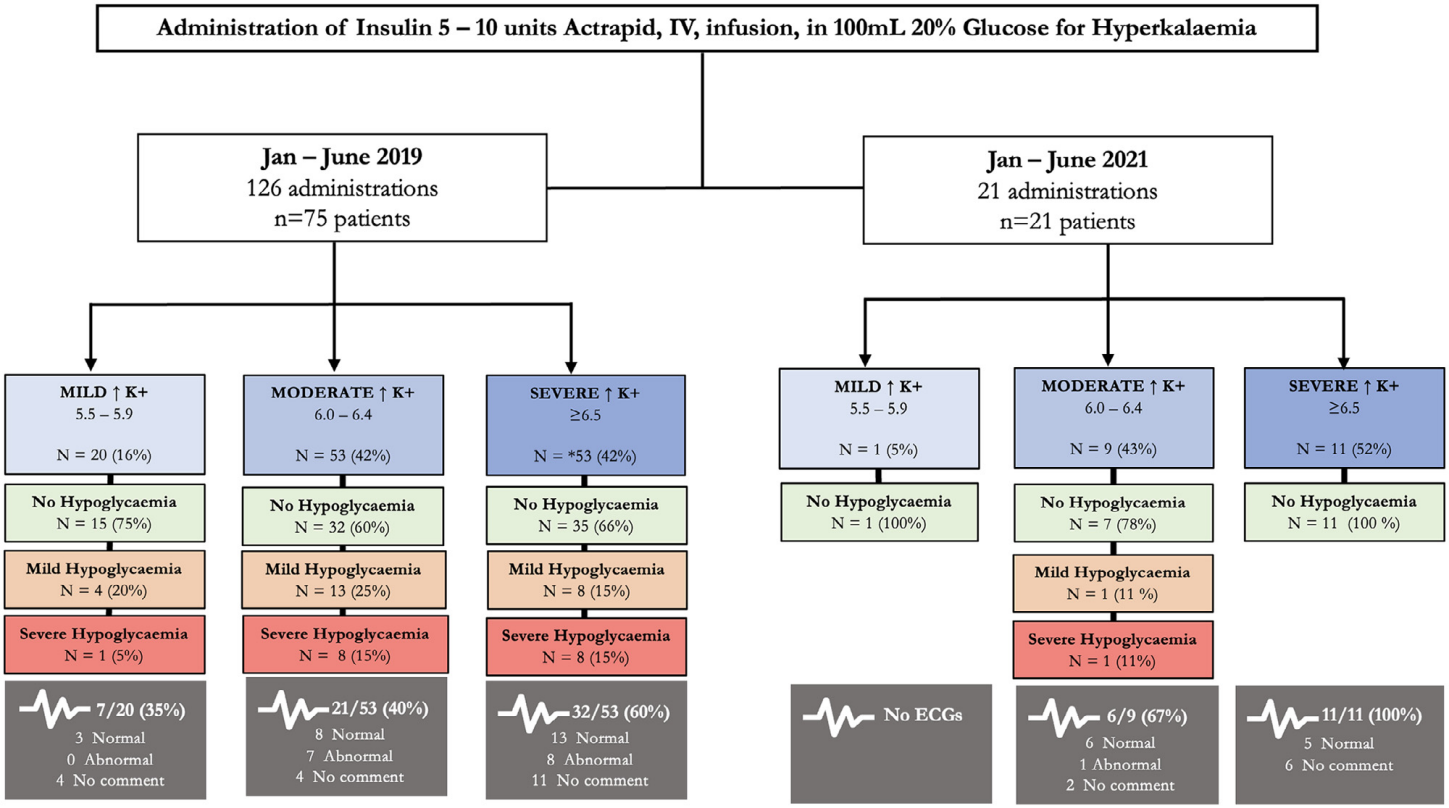
Study flow chart showing the breakdown of insulin/dextrose treatments for mild, moderate and severe hyperkalaemia, incidence of hypoglycaemia in the first 24 h following insulin/dextrose and whether ECG was recorded (grey boxes). *Two patients did not have their blood sugar levels monitored/recorded post-insulin/dextrose treatment.

*Abbreviations: ↑ K; hyperkalaemia.*

In 2019, insulin was frequently being administered inappropriately (outside the guideline indications for treatment), 58% (73/124) of patients had a potassium ≤6.4 mmol/L; only 38% (28/73) of these had an ECG, of which 25% (7/28) were abnormal. In contrast, 44% (9/21) had had a potassium ≤6.4 mmol/L in 2021, with a greater proportion of these administrations 67% (6/21) having a recorded ECG (Fig. 2).

### Sodium zirconium cyclosilicate use in hyperkalaemia

**Fig. 3:**
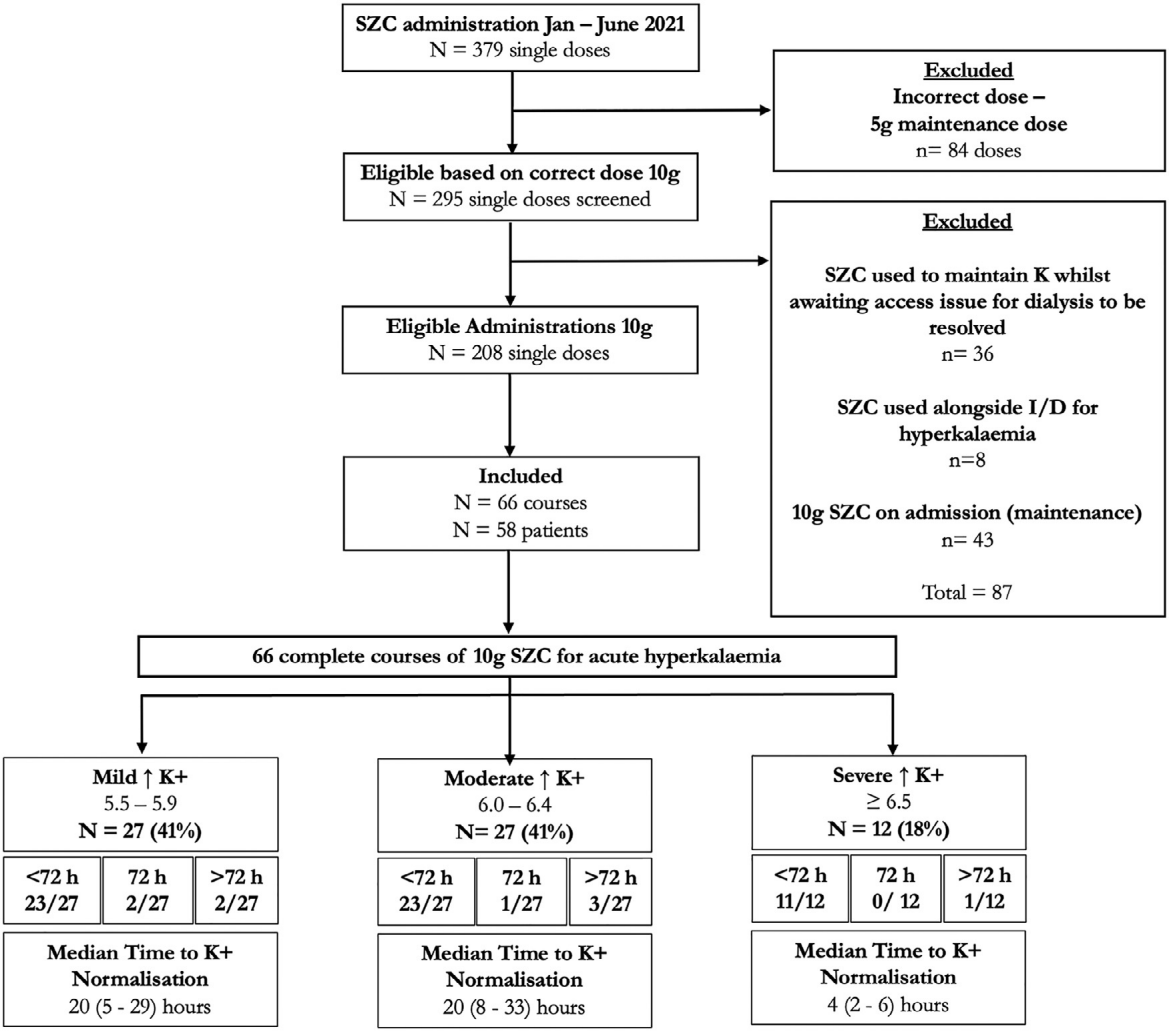
Flow diagram showing the search outcomes, eligible patients, reasons for exclusion and patients included in the final analysis for SZC use for acute hyperkalaemia. Breakdown of SZC use for acute hyperkalaemia length of treatment, and median time to potassium normalisation.

*Abbreviations: SZC;* Sodium zirconium cyclosilicate*, ↑ K; hyperkalaemia, I/D; insulin/dextrose, h; hours*

The reduction in insulin use in 2021 was associated with a rise in the use of SZC, for which there were 66 treatment initiations, (varying in length with a total of 208 single administrations in 58 patients) for the acute treatment of hyperkalaemia (Fig. 3). In 53 of 66 SZC administrations, at least one blood sugar was recorded within 24 h of starting treatment. In those, only one patient had a recorded hypoglycaemic episode, in which the blood glucose dropped to 2.8 mmol/L.

Iatrogenic hypokalaemia (K < 3.5) during or within 72 h of stopping SZC occurred in 10/66 SZC treatment courses. Median time to lowest potassium level, amongst those experiencing hypokalaemia, was 34 (6–63) h. In those who experienced hypokalaemia, 60% (6/10) of patients were diabetic and 60% (6/10) were treated with dialysis. During the treatment course with SZC, there were no reported cardiac arrests, QT prolongation or significant GI disturbances.

The likelihood of developing hypoglycaemia, in those treated for acute hyperkalaemia, was significantly greater in patients treated with insulin/dextrose and those treated in the initial observation period 2019 (Table 3). There was no evidence of a difference in sex, age, history of diabetes or being on dialysis. Following multivariate logistic regression, treatment in 2019 as opposed to 2021 was independently predictive of hypoglycaemia (OR 4.9 [1.3–31.8] *P* = 0.03).

**Table 3:** Risk factors for hypoglycaemia in patients being treated for acute hyperkalaemia.

Variable	Univariate		Multivariariabe	
	Unadjusted OR (95% CI)	*P* value	Adjusted OR (95% CI)	*P* value
**Variable included**				
Treatment (insulin/dextrose)	11.3 (3.3–70.6)	*0.011	2.7 (0.3–23.7)	0.34
Treatment period, 2019 (vs 2021)	9.1 (3.5–31.2)	*<0.001	4.9 (1.3–31.8)	*0.03
**Variable excluded**				
Age (>65 years)	1.0 (0.5–2.0)	1.0		
Sex (male)	1.6 (0.8–3.2)	0.2		
Diabetes mellitus	1.3 (0.7–2.6	0.5		
On dialysis	0.8 (0.4–1.6)	0.6		

Risk factors for hypoglycaemia based on 154 patients and 213 treatments for hyperkalaemia in the two study periods.

## Discussion

The baseline data demonstrate that there is a considerable incidence of iatrogenic hypoglycaemia that arises from the use of insulin/dextrose for the management of hyperkalaemia in patients with renal disease. With the two-fold introduction of SZC alongside changes in patient care after the administration of insulin/dextrose (including hourly blood glucose monitoring for 6 h), we observed more appropriate use of insulin/dextrose, as well as a significant reduction in the iatrogenic burden of hypoglycaemia.

While the morbidity associated with insulin/dextrose has been demonstrated previously, this QIP was able to demonstrate a ‘real-world’ reduction in iatrogenic hypoglycaemia secondary to insulin/dextrose, through simple interventions and the introduction of SZC.

Previous attempts to reduce the morbidity associated with in insulin/ dextrose have looked at altering the administration of insulin. A recent scoping review of anti-hypoglycaemic strategies demonstrated no differences in the prevalence of hypoglycaemia when comparing insulin dose (<10 units vs ≥10 units), rate of insulin administration (continuous vs bolus), type of insulin (regular vs short-acting) or timing of insulin administration relative to dextrose.^1^

The incidence of hypoglycaemia in this QIP was higher than the 6–21% incidence reported in the literature;^3^ this may relate to differences in clinical settings, protocols for hyperkalaemia treatment as well as the high prevalence of patients with impaired renal function in populations studied.^8^^,^^9^ Renal dysfunction increases the risk of hypoglycaemia via reduced insulin clearance, decreased gluconeogenesis during uraemia and increased glucose uptake of red blood cell during haemodialysis.^3^^,^^8^^,^^10^ Furthermore, definitions of hypoglycaemia differ in the literature; one study reported a 13% incidence of hypoglycaemia in 221 inpatients with end-stage renal disease; however, the authors defined hypoglycaemia as blood glucose <3.33 mmol/L.^7^

Given that hypoglycaemia occurred at a delayed time point, capillary blood glucose should be monitored hourly for a minimum of 6 h after insulin/dextrose administration. This is in keeping with the recommendations of the UK Kidney Association (UKKA).^2^ Staff education about the importance of blood glucose monitoring following insulin/dextrose may have prompted them to ensure all steps taken to ensure insulin/dextrose is being initiated appropriately, i.e. after ECG changes seen. There is heterogeneity in previous studies in time to hypoglycaemia; a recent review of 62 studies reported median timing of 124 min (interquartile range 102–168).^1^ Such differences may arise due to differing patient populations and glucose-monitoring protocols. It is also important to balance hypoglycaemia risk with patient discomfort and staff capacity for regular capillary blood glucose monitoring. While people without diabetes have been identified as an ‘at risk’ population from insulin-related hypoglyceamia,^6^^,^^8^ this was not demonstrated in this study (Table 3). Treament in 2019 (pre-intervention) vs 2021 independently predicted hypoglycaemia. It is likely that the introduction of this simple post-treatment intervention contributed to staff awareness and overall reduction in incidence of hypoglycaemia.

The initial audit demonstrated that a large number of patients inappropriately received insulin/dextrose for potassium levels ≤6.4 mmol/L, in the absence of cardiac rhythm abnormalities. Understandably, the ECG can be normal in severe hyperkalaemia as the association of hyperkalaemia with arrythmias is dependent on factors including the rate of rise of serum potassium, red blood cell concentration (reflecting level of potassium at tissues), pH and calcium concentration, all of which can be difficult to appreciate in the acute setting, potentially leading to physician uncertainty and tendencies to prescribe insulin/dextrose.^11^^,^^12^

In the initial observation period of the QIP, 64% (81/126) of insulin/dextrose administrations were delivered overnight, between 21:00 and 09:00; 50% of the patients who received insulin/dextrose were dependent on haemodialysis for renal replacement therapy. Despite the availability of haemodialysis out-of-hours, delivering dialysis can be logistically challenging, and highlights the need for alternative pharmacological agents to aid physicians’ management of moderate hyperkalaemia. The significant uptake of SZC in this QIP was seen alongside a significant reduction (P < 0.0001) in the use of insulin/dextrose, despite a comparable number of admissions between the two observation periods. The overall reduction of insulin/dextrose post-intervention may be driven by the education delivered and the empirical monotherapy alternative with SZC. The efficacy of SZC in the reduction of potassium has been demonstrated in >1,700 patients.^13^ A 10 mg dose of SZC reduces potassium by 0.11 mmol/L within 1 h and by 0.73 mmol/L by 48 h compared to placebo. Serum potassium reduction was dose dependent, with greater effects seen at higher baseline potassium levels.^14^ For patients on the borderline of requiring dialysis, these modest potassium reductions may be of clinical value.

This QIP provides support for the UKKA guideline, which recommends that SZC is considered in the acute management of moderate hyperkalaemia (serum potassium 6.0–6.4 mmol/L) in the abscence of ECG changes.^2^ Unlike previous studies, which have demonstrated the benefits of SZC in conjunction with insulin/dextrose, this QIP sheds light on its use as a sole agent in acute moderate hyperkalaemia.^14^ SZC had a minimal side effect burden in our population and may be better tolerated than alternatives such as calcium resonium, which is associated with significant gastrointestinal side effects.^9^ SZC also offers the advantage of reduced nursing time to admnsiter intravenous insulin/dextrose. While none of the patients studied experienced significant cardiac arrythmias, we report a slightly higher hypokalaemia incidence of 15% compared to the 4.1% reported in other studies.^2^ The lower incidence in other studies may be due to the treatment of chronic hyperkalaemia with lower doses of SZC. Iatrogenic hypokalaemia is a risk associated with SZC use;^2^ in this study, the 15% burden of hypokalaemia in our study population occurred on average around 34 (6–63) h following administration. Our data highlight the need for close monitoring following the initiation of SZC to ensure early recognition of this side effect.

## Limitations

This is single-centre QIP with a high proportion of patients with advanced renal impairment. Those with severe renal disease or on renal replacement therapy may be at increased risk of hypoglycaemia, thus limiting the generalisability of this study. Furthermore, the reduction in hypoglycaemia we observed following insulin/dextrose may have been influenced by other factors such as patient-related factors. Point of care blood glucose measurements can be frequently erroneous in the critical range. As a retrospective study, the results may have been skewed by multiple confounders, such as the varying insulin doses that were used by clinicians. To enhance the generalisability of our findings, it would be beneficial for future studies to assess the effectiveness of our recommendations and guidelines in individuals with normal renal function. Additionally, to strengthen the validity of our conclusions and mitigate potential confounding factors, it would be valuable to conduct prospective studies featuring standardised timing for blood glucose measurements, consistent methods for blood glucose monitoring, and uniform treatment regimens with both insulin/dextrose and SZC. There was also limited information on hospital stay, long-term patient outcomes as well healthcare experience with the interventions used. A cost-effectiveness analysis would have further strengthened the intervention delivered in this QIP. Nonetheless, NICE states that the ‘The cost-effectiveness estimates for sodium zirconium cyclosilicate suggest that it is a good use of NHS resources for treating acute life-threatening hyperkalaemia in emergency care’.^9^ Our guideline suggests 10 units of insulin for hyperkalaemia, but we found that ward practices vary. Real-world studies like this highlight clinical variability, emphasising the importance of observational data.

## Conclusion

Significant harm arises from the use of insulin/dextrose for the management of hyperkalaemia in patients with renal disease. The two-fold introduction of SZC alongside changes in patient care after the administration of insulin resulted in more appropriate use of insulin/dextrose, as well as a significant reduction in the iatrogenic burden of hypoglycaemia.

## Funding

The renal department is supported by the 10.13039/100006662NIHR Biomedical Research Centre.

## Author contributions

**SAS;** Conceptualization, Data curation and analysis, Methodology, Writing- Original Draft, Editing **IK;** Conceptualization, Data curation and analysis, Methodology, Review. **HJ;** Conceptualization, Data curation, Methodology; Review. **SB;** Data curation, Review. **RB;** Data curation, Methodology; Review. **JL;** Conceptualization; Review. **DT;** Conceptualization; Review. **DA;** Conceptualization; Review. **RC;** Conceptualization; Supervision; Validation; Writing - Review and Editing.

## Declaration of competing interest

None of the authors have any conflict of interest or relationships with industry that could have influenced this manuscript.
